# Supplementary material for: Pathways to psychiatric care in Debre Berhan, Ethiopia: A cross-sectional study
Source: PLoS One. 2025 Jul 24;20(7):e0328724. doi: 10.1371/journal.pone.0328724 (PMC12289035; doi:10.1371/journal.pone.0328724)
Supplement: Attachment — Submitted filename: responce to authors.docx [file pone.0328724.s003.docx]

**Title**

More of the manuscript framed the title to" indirect path psychiatric care". So should modify it.

**Abstract**:

Methodology: should explain study period and major components are missed. So should rewrite it.

1. What does mean? “We conducted a 25 multivariable binary logistic regression analysis”

2. Key words: add the word “factors”

**Introduction**

This article aims to provide a comprehensive understanding of indirect path psychiatric care. The introduction statements should be revising to reduce unnecessary sentences, and summarized again. Additionally, grammatical should be corrected to ensure a smooth reading experience. There is no sufficient introduction and explanation about the topic area.

**Objective:** Not clearly set in the manuscript.

**Materials and methods**

1. Not clear enough

2. The study setting mental health service types is not clear.

3. Should mention the types of mental health services in the DCSH and explain the average number of psychiatric patients (visits) in every month in the DCSH.

3. Study period Format should be rewrite or stated like **Date/month/year**.

4. Franckly, sample size calculation is not clear.

5. Both Sampling procedures and sampling techniques are not clear.

6. Eligibility criteria is not clear and Write briefly about the inclusion and exclusion criteria.

7. How many patients were there during the time frame of the study? How many of these patients were not included in the study? Explain clearly why they were not included. If not, it’s difficult to accept the study.

8. Show clearly how it was possible to achieve the actual sample size i .e 454 out of 828 patients in three months.

9. Show clearly how many new cases and follow up cases were during the study period?

**Study tools: clear**

**Source populations:** not clear enough	**Study populations?**

**Operational definition is not clear**

VIF: Write down the value of VIF in number how much it was and also write down the scientific explanation of VIF.

**Model of fitness**...Not clear, how much it was.

The manuscript **has no ethical section**. Since its human participant research you should have incorporate the ethical issues in the manuscript.

**Result part**

1. When the clinical variables were categorized, the authors used the term "**psychosis**" as a disorder category. This is not a disorder category rather its symptom. So what do you mean “**Psychosis**" in your study or use the correct disorder category based on ICD-11 or DSM-5 diseases classification manuals.

2. In the associated factors table the P **value is not set.**

**Discussion part**

1. Should incorporate the clinical implication of the study finding?

2. The focus of the recommendations should be specific on the study findings.

Example...should recommend about age, being female, and being farmer.
