# Supplementary material for: Pathways to psychiatric care in Debre Berhan, Ethiopia: A cross-sectional study
Source: PLoS One. 2025 Jul 24;20(7):e0328724. doi: 10.1371/journal.pone.0328724 (PMC12289035; doi:10.1371/journal.pone.0328724)
Supplement: Attachment — Submitted filename: Response to Reviewers.docx [file pone.0328724.s004.docx]

**Response to Academic Editor**

1. Be consistent with terms like informal, indirect, etc.

*Thank you for the comment. We have made a correction.*

2. Please include additional sentences that show the gap of the study in the introduction part abstract.

*Thank you for the comment. We understand that the introduction section is slightly shorter due to word count limitations and the need for brevity. However, we have now attempted to highlight an additional research gap. Please refer to page 2, lines 17-18 (also reported below).*

Previous studies in Ethiopia have shown that PWMDs often prefer indirect sources of help, and many experience a long duration of untreated illness.

3. Please include additional information like sampling techniques, response rate, etc in the methodology part of the abstract.

*Thank you for the comment. We understand that the methods section is slightly shorter and lacks some important points due to word count limitations and the need for brevity. However, we have now attempted to rewrite it. Please refer to page 2, lines 24-28 (also reported below)*

A cross-sectional study was conducted from March 30 to May 30, 2021, enrolling 446 psychiatric patients at Debre Berhan Hospital. The response rate was 98.2%, and the sampling technique used was consecutive. Data were collected using the WHO Encounter Form and analyzed. Variables with a p-value of <0.25 in bivariable analysis were entered into multivariate binary logistic regression, with statistical significance set at p < 0.05.

4. Currently the focus of mental health treatment is to consider primary care, including traditional healing, to reduce the treatment gap, particularly for low-income countries, but you are recommending the indicated pathway as a negative; please acknowledge such approaches.

*We would like to thank the editor for raising this important issue. We have now revised the manuscript, particularly our recommendations, to incorporate/ integrate indirect pathways with modern psychiatric care. Please refer to page 36, line 469-488 for more details.*

5. Please proofread the manuscript for English language and grammar.

*Thank you for the comment, we did that.*

6. Please be specific with your study populations (which type of patients) since there are different types of people with symptoms of mental illness.

*Thank you for the comment. We have made efforts to manage it appropriately.*

7. Was the tool validated within a similar population?

*Thank you for the comment. The WHO encounter form is a tool that has been utilized in different parts of Ethiopia to assess pathways for PWMDs. Prior to commencing this study, we conducted a pretest to ensure its suitability. The tool has also been validated in similar populations to guarantee its effectiveness and relevance for our research.*

8. Please interpret your result in terms of other studies, policies, and guidelines.

*Thank you for the comment. We have attempted to interpret our findings in terms of other studies.*

**Response to Reviewer #1**

**Dear Authors,**

The study you undertake provides valuable insights into the factors influencing pathways to psychiatric care in Debre Berhan, Ethiopia. The authors' comprehensive analysis of sociodemographic characteristics, perceived stigma, diagnosis, and help-seeking behaviors contributes to a better understanding of the challenges faced by individuals with mental health disorders in accessing appropriate care. Below are some concerns you need to go through and react

**Introduction part**

**Concerns**

- **Line 102-103:** You mention that little is known about pathways to psychiatric care in Ethiopia, especially indirect pathways. Could you elaborate on the research gap and why this is a significant area to investigate?

*Thank you for bringing this into our attention. We have now added articles that shows why it is important to investigate* pathways to psychiatric care. *Please see page 6, lines 98-100 of the revised manuscript (also reported below).*

Most of the studies in Ethiopia are about help-seeking behaviour (11, 23, 25-31), which is more about looking the source that PWDs gone through rather than explicitly identifying the first point of contact as is done in pathways to care.

- **Line 111-112:** How might the availability of holy water places influence the decision-making process of PWMDs in Ethiopia? Are there any specific beliefs or practices associated with these places that might impact their choice of care?

*Thank you for bringing this into our attention. We have now added articles that shows how the availability of holy water places and specific beliefs or practices influence the decision-making process. Please see page 7, lines 111 – 114 of the revised manuscript (also reorted below).*

In Ethiopia, many PWMDs seek holy water for healing, believing their illness has spiritual causes. This delays psychiatric care and limits use of medical services. Stigma and poor access to mental health care further encourage reliance on religious rituals as treatment (35, 36).

- **Line 116-118:** How did the WHO pathways to psychiatric care encounter form contribute to the data collection and analysis process? What specific insights did it provide that might not have been possible with other methods?

*Thank you for the question. The WHO pathways to psychiatric care encounter form significantly contributed to data collection and analysis by providing a structured and comprehensive framework for documenting the patient's journey to psychiatric care. By capturing four major pathway contacts, including the initial help-seeking source and subsequent contacts, along with 22 detailed items, the form offered critical insights into the sequence of events leading up to care. This design enabled the identification of delays, referral patterns, and sources of initial help, whether from family, friends, or healthcare professionals. Such insights would be difficult to capture through interviews or general surveys, as the form’s structured format ensures a clear record of each stage of care. The focus on time delays and the types of referrals also revealed systemic barriers and potential inefficiencies within healthcare systems that could be improved. Moreover, the form allowed for a detailed understanding of how patients navigate different contacts, which could help inform interventions and policies aimed at reducing delays in care. By documenting the flow from the first point of contact to the final treatment stage, the encounter form provided invaluable data that could drive improvements in psychiatric care accessibility and help optimize the pathway for future patients.*

**Method part**

**Concerns**

- **Line 134:** Could you elaborate on the reason for using a 3% margin of error and a 95% confidence interval? Were these values chosen based on specific guidelines or conventions in your field?

*Thank you for the question. We used a 3% margin of error to obtain a relatively large sample size for greater precision. The 74% proportion was derived from a previous study and was used to determine the sample size. The choice of a 3% margin of error was based on this proportion, ensuring a more accurate estimate within the study’s context.*

- **Line 138:** How was the expected number of PWMDs receiving psychiatric care at DBCSH during the study period obtained? Was this based on previous data, projections, or other sources?

*Thank you for your question. During the two-month study period, 828 PWMDs including both follow-up and new patients were expected to receive psychiatric care at DBCSH. The clinic typically serves an average of 400 to 500 PWMDs per month. The estimated number of patients (828) was obtained through consultation with the head of the psychiatry department and by reviewing records from previous and corresponding months of the prior year.*

*Using a consecutive sampling technique, 454 participants were initially enrolled. After non-responses, the final sample size was 446.*

*Patients were included in the study if they were above 18 years of age, able to communicate effectively, and capable of providing informed consent. Those who were under 18, had cognitive impairments, or experienced communication difficulties that hindered informed consent and participation were excluded.*

- **Line 140:** Please provide more details about the non-response rate used for the final sample size calculation. How was this rate determined?

The non-response rate was determined by calculating the percentage of participants who did not complete the interview out of the total approached for participation. We initially targeted 454 PWMDs, but eight individuals did not complete the study, three interrupted the interview, and five declined participation. This resulted in a non-response rate of ***1.8%*** (8/454), leading to a final sample size of ***446*** with a ***98.2% response rate***. The final sample size was based on actual responses rather than the total approached.

- **Line 142:** Can you explain why consecutive sampling was the choice for your research, considering its non-probability characteristics?

*Thank you for your comment. We acknowledge the limitations of non-probability sampling; however, we chose consecutive sampling due to the short study period of only two months. This approach allowed us to efficiently recruit all eligible participants who met the inclusion criteria within the given timeframe, ensuring a feasible and systematic data collection process*

- *.***Line 145:** Could you elaborate on the three-item questionnaire used to assess participant capacity to consent? What were the specific questions asked?

*Thank you for the comment. We have elaborated the questionnaire, please refer page 9, and line 152-155 (also reported below).*

Before enrollment, we assessed participants’ capacity to consent using a three-item questionnaire. After explaining the purpose of the study, the risks of participating, and the benefits of participating, we asked the participants the same three questions. If they correctly responded to at least two out of three (if the participant scored three and above out of six) (37), we obtained their consent to participate in the study.

- **Line 147:** Given the potential for bias in verbal consent, consider discussing any measures taken to ensure the validity and reliability of the consent process.

*Thank you for the question. To ensure the validity and reliability of the verbal consent process and minimize potential bias, we implemented several measures. First, we provided a standardized explanation of the study’s objective, risks, and benefits to all participants, ensuring consistency. We also informed participants of their right to decide whether to enroll and their ability to discontinue participation at any time. Second, we incorporated a comprehension check by asking three key questions about the study. Only those who correctly answered at least two out of three were eligible to provide consent. Third, we assured confidentiality and adhered to data security requirements by not recording participants' names or identifiable information. All collected data was stored on a password-protected personal computer accessible only to the principal investigator. Additionally, interviews were conducted in a private room to ensure confidentiality and privacy. These steps helped ensure an informed, voluntary, and ethically sound consent process.*

- **Line 159:** Given that the WHO encounter form has been used in previous studies in Ethiopia, what are the specific challenges or limitations that you anticipate encountering in using this tool in your study?

*Thank you for the question. The WHO Encounter Form has been used in previous studies in Ethiopia, but we anticipate different challenges in using it. First, it tracks the healthcare-seeking pathways of PWMDs, but patients may struggle to recall the exact sequence of institutions they visited. Memory recall issues could affect data accuracy. Second, the form does not allow participants to provide detailed explanations of their long and complex care pathways, limiting qualitative insights.*

- **Line 174:** Could you provide more details about the specific previous studies that you adapted the questionnaires from for assessing social-related factors? How did you ensure that the adapted questionnaires were appropriate and valid for your study population?

*Thank you for the questions. To clarify, we used both the Jacoby 3-item stigma scale and the Oslo 3-item social support scale in our study. These tools were utilized to assess perceived stigma and social support, respectively, and to understand the social-related factors associated with psychiatric care pathways. Both tools were chosen based on their use in previous studies. To ensure their appropriateness for our study population, we conducted a pretest and validated the instruments accordingly.*

- **Line 187:** Why did you choose to develop your questionnaire to assess clinical-related characteristics, rather than using existing validated instruments? What were the specific factors that you wanted to measure that were not covered by the WHO encounter form?

*Thank you for your question. We could not find a validated tool that directly addressed our clinical questions. As such, we developed an eight-item questionnaire specifically to assess clinical-related variables not covered by the WHO encounter form, such as current diagnosis, symptoms, type of visit, and history of psychiatric consultation. Some data, like diagnosis, were collected from patient charts, while other information was gathered using items we developed to capture factors we identified as important in understanding pathways to care. This approach allowed us to gather more targeted data that was crucial for our study.*

- **Line 193:** Could you provide more information about the specific items from the SRQ-20 that you included in your study? How did you ensure that these items were relevant and appropriate for assessing comorbid somatic symptoms in your population?

*Thank you for the questions. As stated in line 205 -208, we included items from the SRQ-20 to assess comorbid somatic symptoms frequently reported in PWMDs. Specifically, we selected items that address symptoms like headache, abdominal pain, back pain, as well as suicidal ideation and attempts. To ensure these items were relevant and appropriate for our study population, we conducted a pretest and also reviewed the existing literature and consulted experts in the field, ensuring the items align with the clinical presentation of PWMDs and the objectives of our research. This approach ensured both content validity and relevance to our study.*

- **Line 200:** What specific mental health conditions were included in the assessment of mental health literacy?

*Thank you for the question. The mental health literacy section of our study assessed participants' awareness of the cause, severity, treatment, and places of care for mental health problems. We used a ten-item questionnaire to evaluate these aspects, which were not covered by the WHO pathways to psychiatric care encounter form. The items were adapted from previous studies in Ethiopia (13, 33). The questionnaire focused on participants' perceptions of mental illness in general, rather than specific mental health conditions. It aimed to assess their views on the causes, treatment options, and curability of mental health issues.*

- **Line 202:** Can you provide more details about the WHO pathways to psychiatric care encounter form and how it differs from the ten-item questionnaire?

*Thank you for your question. The ten-item questionnaire used in our study is adapted from previous Ethiopian studies, specifically designed to assess mental health literacy. It is not the same as the Ten-Item Questionnaire (TIQ), which is a brief screening tool for assessing prior psychiatric service use. Our ten-item tool, on the other hand, focuses solely on evaluating participants' awareness of mental health issues. The WHO pathways to psychiatric care encounter form, in contrast, focuses on tracking the care-seeking journey of individuals, addressing their specific healthcare encounters.*

- **Line 203:** Please elaborate on the specific studies from Ethiopia (references 13 and 29) that were used to adapt the ten-item questionnaire. What were the key similarities and differences between those studies and your research?

*Thank you for your question. The similarity between our study and the studies cited (references 13 and 33) is that all involved PWMDs receiving care at hospital settings, and all utilized similar tools such as the WHO encounter form. However, the key differences lie in the settings and timeframes: the previous studies were conducted in Mekelle and Jimma over a decade ago, during a time when psychiatric care was less accessible and the number of mental health professionals was limited. In contrast, our study was conducted in a context where, over the past ten years, the Ministry of Health has implemented strategies to improve access by decentralizing mental health services and training primary care workers in basic psychiatric care. Please refer page 6 , line 103 for more details*

- **Line 204:** How did you ensure the ten-item questionnaire adequately assessed participants' perceptions of the cause, treatment, and curability of mental illness? Were there any pilot testing or validation procedures conducted?

*Thank you for the question. To ensure the ten-item questionnaire adequately assessed participants' perceptions of the cause, treatment, and curability of mental illness, we conducted a pretest and adapted the items from previous studies in Ethiopia. We reviewed existing literature and consulted experts in the field to confirm that the items were relevant and appropriate for our study population. The pretest allowed us to validate the items further and ensure they captured the essential aspects of mental health literacy, including cause, treatment options, and perceived curability of mental illnesses.*

**Result part**

- In **lines 222-225**, you report a high response rate of 98.2% among PWMDs. Could you please provide more details about the characteristics of the non-respondents (e.g., age, gender, disability type) to assess potential biases in your sample? Additionally, could you elaborate on the reasons for the interruptions and unwillingness to participate, as this may shed light on factors influencing participation rates?

*We would like to thank the reviewer for raising this important issue. We have now added the characteristics of the non-respondents and the reasons for the interruptions and unwillingness to participate, please refer page, line 244-249 (also reported below).*

We included 446 PWMDs in the study, achieving a 98.2% response rate. Out of the 454 individuals approached, eight did not complete the interview. Five of them were female, aged between 22 and 40 years. Reasons for non-response included interview interruption (three participants) and unwillingness to participate (five participants). Some did not provide specific reasons, while others cited time constraints despite being informed of the interview length. A few participants also exhibited anxiety, which may have influenced their decision to discontinue.

- **Line 285**: Could you elaborate on why the adjusted odds ratios for significantly associated variables are substantially higher than their crude counterparts?

*Thank you for the question. The adjusted odds ratios (AORs) for significantly associated variables are substantially higher than their crude odds ratios (CORs) due to the control of potential confounding factors in the multivariable analysis. The increase in AORs suggests that certain variables, such as stigma (AOR: 5.86 vs. COR: 2.84) and being a farmer (AOR: 13.00 vs. COR: 10.56), had stronger associations with indirect pathways to psychiatric care after adjusting for other influencing factors. This indicates that confounders in the crude analysis may have masked the true strength of these associations.*

**Discussion part:**

- **Lines 423-424**: Could the authors elaborate on the potential mechanisms through which perceived stigma, family influence, and lack of access contribute to the decision to utilize indirect pathways?

*We would like to thank the reviewer for raising this important issue. In the revised manuscript, we have added more information about how perceived stigma, family influence, and lack of access contribute to the decision to utilize indirect pathways. Please refer page 35, line 450-456(also reported below).*

Stigma leads individuals to avoid psychiatric care due to fear of discrimination, opting instead for traditional or religious healers. Family members, shaped by cultural beliefs, may direct individuals away from psychiatric services, attributing symptoms to supernatural causes. Additionally, barriers such as financial constraints, geographic distance, and limited mental health infrastructure result in delays and reliance on non-medical interventions before seeking psychiatric treatment. Addressing these factors is crucial for improving timely access to care.

- **Line 445**: Given the prominent role of religious healers in this study, how do the authors envision integrating these practices with modern psychiatric services to improve access to care?

*Thank you for raising this important point. We have now added some recommendation how the integration improve access to care. Please refer page 36, line 478-482(also reported below).*

Integrating religious healers with modern psychiatric services through respectful collaboration, joint training, and culturally sensitive education can improve awareness, timely referrals, and reduce stigma. Embedding services in faith based settings enhances accessibility, encourages early intervention, and bridges traditional beliefs with evidence based care, ultimately improving outcomes for PWMDs.

- **Lines 443-447**: Beyond the recommendations for collaboration between mental health professionals and religious organizations, what other policy interventions could be implemented to address the challenges identified in this study?

*Thank you for this comment. We have now added a statement on how policy interventions could address the identified challenges. Please refer page 36, line 482-486(also reported below)*

This study has implications for healthcare providers, caregivers, PWMDs, researchers, and policymakers. For family members, it emphasizes the importance of maintaining close relationships with PWMDs to address self-stigma and promote direct support. Researchers can use this study as a foundation for future community-based studies to better understand these pathways. Policymakers could focus on fostering collaboration between mental health professionals and religious organizations, facilitating referrals to modern psychiatric services while integrating religious therapy. Enhancing psychiatric services, expanding community access, and merging modern care with traditional healing practices are crucial steps. Respectful collaboration, joint training, and culturally sensitive education can improve awareness, encourage timely referrals, and reduce stigma. Embedding services in faith-based settings can increase accessibility, support early intervention, and bridge traditional beliefs with evidence-based care, improving outcomes for PWMDs.

Additionally, expanding psychiatric services at the community level, strengthening mental health infrastructure, training more professionals, and integrating services into primary care can reduce treatment delays and ensure equitable access for people with mental disorders. Mental health professionals and policymakers should consider focused interventions for older people, individuals with low educational levels, farmers, and females, as these groups are more likely to choose indirect pathways to care. Targeting these groups with tailored interventions can help bridge gaps in care, improve access to mental health services, and ensure more equitable treatment for diverse populations.

**Better add the followings**

1. Study strengths and limitations

Thank you for the comment. We have now added *the study's strengths and limitations are already included in the manuscript. Please refer to page 37, line 487(also reported below)*

Strength and limitation study

This study is among the first in Ethiopia to explore factors associated with using indirect pathways to psychiatric care, whereas previous studies mainly addressed delays in help-seeking. We used standardized tools, including the WHO encounter form, Oslo Social Support Scale, and the 3-item Jacoby Scale.

However, the findings should be interpreted with caution. As the study was conducted at the hospital level, it may not reflect the community-wide care-seeking pathways. The cross-sectional design limits causal inference, and recall bias may be present due to the nature of the illness and long illness durations. To mitigate this, we provided clear instructions, adequate time, and assessed participants' capacity to respond. Social desirability bias is also possible, though we minimized this by reassuring participants and conducting interviews post-care.

2. Recommendation

*Thank you for your recommendation. We have incorporated recommendations within the discussion section, following each associated factor.*

**Response to Reviewer #2**

**Title**

More of the manuscript framed the title to" indirect path psychiatric care". So should modify it.

*Thank you for your comment. We have titled the study Pathways to Psychiatric Care because the tool we used, the WHO Encounter Form, evaluates both direct and indirect pathways. Given this comprehensive approach, we believe the current title accurately reflects the study's scope and objectives. Therefore, we propose to proceed with the existing title.*

**Abstract**:

Methodology: should explain study period and major components are missed. So should rewrite it.

*Thank you for raising this comment. We have now rewrite the methods section, please refer page number 2, and line 24*

*A cross-sectional study was conducted from March 30 to May 30, 2021, enrolling 446 PWMDs at DBCSH. The response rate was 98.2%, and the sampling technique was consecutive. Data were collected using the WHO Encounter Form and analyzed. Variables with p < 0.25 in bivariable analysis were entered into multivariate binary logistic regression, with significance set at p < 0.05.*

1. What does mean? “We conducted a 25 multivariable binary logistic regression analysis”

*Thank you for the question. To clarify, we entered 25 variables into the multivariate binary logistic regression based on a p-value of <0.25 from the bivariable analysis. This approach allowed us to examine the variables that were potentially significant in predicting pathways to psychiatric care.*

2. Key words: add the word “factors”

*Thank you for raising this issue. We have incorporated the word as requested in the manuscript. Please refer to page 3, line 42. Here is the corrected part:*

**Keywords:** Pathways, direct and indirect pathways, people with mental disorders, determinants, factors, psychiatric care, Ethiopia.

**Introduction**

This article aims to provide a comprehensive understanding of indirect path psychiatric care. The introduction statements should be revising to reduce unnecessary sentences, and summarized again. Additionally, grammatical should be corrected to ensure a smooth reading experience. There is no sufficient introduction and explanation about the topic area.

*Thank you for the comment. We have now added statements about indirect pathways to psychiatric care and addressed the grammatical issues. Please refer to page 4, line 46.*

**Objective:** Not clearly set in the manuscript.

*Thank you for the comment. We have clearly stetted the objective of the stud, please refer page 7, and line 119-122(reported below).*

Therefore, this study aims to identify pathways to psychiatric care and factors associated with pathways to psychiatric care among people with mental disorders receiving care at Debre Berhan Comprehensive Specialized Hospital (DBCSH) psychiatry clinic, Debre Berhan, Central Ethiopia, in 2021.

**Materials and methods**

1. Not clear enough

*Thank you for the comment. We have made the necessary corrections based on the specific feedback you have provided below.*

2. The study setting mental health service types is not clear.

*Thank you for the comment. We have now make it clearer, please refer page 8, and line 126-128 (also reported below).*

The study was conducted at the Debre Berhan Comprehensive Specialized Hospital (DBCSH) psychiatric clinic, located in Debre Berhan town, Central Ethiopia. The hospital provides psychiatric services to approximately 5,000 people with mental disorders annually.

3. Should mention the types of mental health services in the DCSH and explain the average number of psychiatric patients (visits) in every month in the DCSH.

*Thank you for the comment. We have made the necessary corrections, please refer page 8, and line 128-133(also reported below).*

The DBCSH psychiatric clinic provides a range of mental health services, including diagnosing and treating both new and follow-up PWMDs. Services offered include pharmacological treatment, psychotherapy, counseling, emergency psychiatric services, and referrals for specialized care. The clinic typically sees an average of 400 to 500 psychiatric patient visits per month, consisting of both new cases and follow-up appointments.

4. Study period Format should be rewrite or stated like **Date/month/year**.

*We would like to thank the reviewer for bringing this into our attention. We have now corrected on page 8, line 133-134 (also reported below).*

We employed an institutional-based cross-sectional study from March 30, 2021, to May 30, 2021.

5. Frankly, sample size calculation is not clear.

*Thank you for the question. We have now clarified the sample size calculation part, please refer page 8, and line 135 (also reported below).*

We used the single population proportion formula to determine the minimum sample size required for this study. The proportion (p) was taken from a previous study conducted in Mekelle, Northern Ethiopia, which reported that 74% of PWMDs utilized indirect pathways (13). The calculation was based on a 3% margin of error and a 95% confidence interval (CI). This yielded an initial sample size of 821 PWMDs.

Since the total source population for this study is less than 10,000, we applied the finite population correction formula to adjust the sample size:

$n$ = $\mathrm{ni}$/ (1 + $\mathrm{ni}$/N)

where:

- n = final minimum sample size
- ni = initial sample size (821)
- N = total source population (expected number of PWMDs receiving psychiatric care at DBCSH during the study period, i.e., 828 PWMDs obtained through personal communication with the department head)

After applying the correction, the adjusted sample size was 413. To account for a 10% non-response rate, the final required sample size was 454 PWMDs.

6. Both Sampling procedures and sampling techniques are not clear.

*Thank you for the comment. We employed a consecutive sampling technique to enroll all eligible PWMDs who visited the psychiatry department of DBCSH during the study period. This non-probability sampling method was chosen to ensure that all eligible participants who met the inclusion criteria were considered until the required sample size was achieved. We have clarified the sampling procedures and sampling techniques on the manuscript based on your feedback. Please refer to page 9, line 149.*

7. Eligibility criteria is not clear and Write briefly about the inclusion and exclusion criteria.

*Thank you for the comment. We have now clarified this please refer to page 9, line 149-158 (also reported below).*

We interviewed all PWMDs receiving care at the psychiatry department of DBCSH who met the eligibility criteria. Inclusion criteria: PWMDs aged 18 and above who could communicate and provide informed consent. Before enrollment, we assessed participants’ capacity to consent using a three-item questionnaire. After explaining the purpose of the study, the risks of participating, and the benefits of participating, we asked the participants the same three questions. If they correctly responded to at least two out of three (if the participant scored three and above out of six) (37), we obtained their consent to participate in the study. Exclusion criteria: PWMDs with severe cognitive impairment or communication difficulties that hindered informed consent and participation were excluded.

8. How many patients were there during the time frame of the study? How many of these patients were not included in the study? Explain clearly why they were not included. If not, it’s difficult to accept the study.

*We would like to thank the reviewer for raising this important issue. During the two-month study period, a total of 828 PWMDs were expected to receive psychiatric care at DBCSH. Of these, 446 participants were included in the study. The remaining 382 patients were not included due to reasons such as not meeting the eligibility criteria (e.g., inability to provide consent, communication difficulties, or being under 18 years old), refusal to participate, or other logistical constraints. The inclusion criteria required PWMDs who could give consent, communicate, and were above 18 years old.*

9. Show clearly how it was possible to achieve the actual sample size i .e 454 out of 828 patients in three months.

*Thank you for the comment. During the two-month study period, 828 PWMDs including both follow-up and new patients were expected to receive psychiatric care at DBCSH. The clinic typically serves an average of 400 to 500 PWMDs per month. The estimated number of patients (828) was obtained through consultation with the head of the psychiatry department and by reviewing records from previous and corresponding months of the prior year.*

*Using a consecutive sampling technique, 454 participants were initially enrolled. After non-responses, the final sample size was 446.*

*Patients were included in the study if they were above 18 years of age, able to communicate effectively, and capable of providing informed consent. Those who were under 18, had cognitive impairments, or experienced communication difficulties that hindered informed consent and participation were excluded.*

10. Show clearly how many new cases and follow up cases were during the study period?

*During the two-month study period, an estimated 828 PWMDs received psychiatric care at DBCSH. The majority were follow-up cases, while 148 were new cases*

**Study tools: clear**

**Source populations:** not clear enough	**Study populations?**

*We would like to thank the reviewer for raising this important issue. All PWMDs receiving care at the DBCSH psychiatry department constituted the source population. The study population included all selected PWMDs receiving care during the study period. We interviewed every PWMD receiving care at the psychiatry department who met the eligibility criteria: being above 18, able to communicate, and capable of providing consent. Before inclusion, we assessed the participant's capacity to consent using a three-item questionnaire. If the participant demonstrated the ability to consent, we obtained verbal consent and conducted the interview.*

**Operational definition is not clear**

*Thank you for the comment. We have clarified the operational definitions, please refer page 10, line 174-180 (also report below).*

Considering the recommendation of the WHO encounter form for developing countries, we classified participants who received psychiatric care directly from modern health care providers such as mental health professionals, general practitioners, or primary health care workers for their current mental health condition as having utilized a direct pathway to care. Conversely, participants who first visited alternative sources such as religious healers, traditional healers, herbalists, or other informal sources before receiving psychiatric care at the DBCSH psychiatric department were classified as having followed an indirect pathway.

**VIF**: Write down the value of VIF in number how much it was and also write down the scientific explanation of VIF.

*Thank you for the question. In our study, the VIF values ranged from* ***1.2 to 3.4*** *indicating that multicollinearity was not a significant concern.*

**Model of fitness**...Not clear, how much it was.

*We assessed the model fitness using the Hosmer and Lemeshow goodness-of-fit test, which resulted in a p-value of 0.062. This indicates that the model fits the data well. We have reported it on page 28, line 316*

The manuscript **has no ethical section**. Since its human participant research you should have incorporate the ethical issues in the manuscript.

*Thank you for the comment. We have included the ethical consideration section, please refer page 13, and line 232 (also reported below)*

This study was conducted after obtaining ethical approval from the ethical review committee of the School of Public Health, Asrat Woldeyes Health Sciences campus, Debre Berhan University. Verbal informed consent was obtained after assessing participants’ ability to consent using a validated three-item tool; those scoring three or more out of six were considered capable (37). Participants were informed about the study's purpose, their right to withdraw at any time, and confidentiality was ensured by conducting interviews in a private setting and securing data on a password-protected computer accessible only to the principal investigator. To prevent COVID-19 transmission, all recommended safety measures were followed, including mask-wearing, physical distancing, and hand sanitization.

**Result part**

1. When the clinical variables were categorized, the authors used the term "**psychosis**" as a disorder category. This is not a disorder category rather its symptom. So what do you mean “**Psychosis**" in your study or use the correct disorder category based on ICD-11 or DSM-5 diseases classification manuals. Schizophrenia and other **psychosis disorder**

*Thank you for the comment. We initially used the term "psychosis" for brevity, but we have now corrected it in the manuscript to "Schizophrenia and other psychotic disorders" in accordance with ICD-11 and DSM-5 classification systems.*

2. In the associated factors table the P **value is not set.**

*Thank you for the comment. Initially, we did not include the P-values for brevity, but we have now incorporated them in the manuscript. Please refer to the updated associated factors table for the inclusion of P-values.*

**Discussion part**

1. Should incorporate the clinical implication of the study finding?

*Thank you your comment. We have now added some clinical implications of the study finding. Please refer page 36, line 469-488(also reported below)*

This study has implications for healthcare providers, caregivers, PWMDs, researchers, and policymakers. For family members, it emphasizes the importance of maintaining close relationships with PWMDs to address self-stigma and promote direct support. Researchers can use this study as a foundation for future community-based studies to better understand these pathways. Policymakers could focus on fostering collaboration between mental health professionals and religious organizations, facilitating referrals to modern psychiatric services while integrating religious therapy. Enhancing psychiatric services, expanding community access, and merging modern care with traditional healing practices are crucial steps. Respectful collaboration, joint training, and culturally sensitive education can improve awareness, encourage timely referrals, and reduce stigma. Embedding services in faith-based settings can increase accessibility, support early intervention, and bridge traditional beliefs with evidence-based care, improving outcomes for PWMDs.

Additionally, expanding psychiatric services at the community level, strengthening mental health infrastructure, training more professionals, and integrating services into primary care can reduce treatment delays and ensure equitable access for people with mental disorders. Mental health professionals and policymakers should consider focused interventions for older people, individuals with low educational levels, farmers, and females, as these groups are more likely to choose indirect pathways to care. Targeting these groups with tailored interventions can help bridge gaps in care, improve access to mental health services, and ensure more equitable treatment for diverse populations.

2. The focus of the recommendations should be specific on the study findings.

Example...should recommend about age, being female, and being farmer.

*Thank you for the comment. We have already included the recommendations, please refer page 36, and line 483-488*
