# Supplementary material for: Pathways to psychiatric care in Debre Berhan, Ethiopia: A cross-sectional study
Source: PLoS One. 2025 Jul 24;20(7):e0328724. doi: 10.1371/journal.pone.0328724 (PMC12289035; doi:10.1371/journal.pone.0328724)
Supplement: Attachment — Submitted filename: Comment by Tamene Berhanu May 9, 2025.docx [file pone.0328724.s005.docx]

**May 8, 2025**

**PLOS ONE**

Pathways to Psychiatric Care in Debre Berhan, Ethiopia: a cross-sectional study

**--Manuscript Draft—**

**Manuscript Number: PONE-D-24-27256R1**

**Reviewed by Tamene Berhanu Alaho (**Mental Health Specialist**)**

**Overall:** I would like to thank for giving chance to review this interesting work. This study will have great value in low and middle income countries to enhance awareness and prevention strategies for mental health wellbeing. This manuscript reviewed strongly by previous reviewers and authors revised manuscript based on comments. I hope this is good job.

I have few minor comments, find it below

**Comments**

**Abstract section**

1. Describe the meaning of pathways to psychiatric care and its impact on mental health

**Introduction/Background**

1. It is better to explain in detail about indirect Pathways to psychiatric care challenges and its contribution to mental disorders.
2. This study mentioned that ‘‘Debre Berhan) is known for the availability of different holy water places, which are considered an alternative source for mental health care. We expect most PWMDs from various parts of the country to visit those places, hoping for a cure’’. What is relevancy for this statement? Holy water place is easily available in most part of Ethiopia. Why various parts of Ethiopian people going to Debre Berhan for hoping cure? Since holy water is similar in any place. How holy water in Debre Berhan is superior than others place to think most part of country’s people to hope cure. Please remove this statement since holy water is common in all regions in Ethiopia, there is no clear evidence for superiority of holy water in different region.
3. Introductions section is bulky. It is better to make short and precise this section.

**Method**

1. Participants for this study from patients receiving care in psychiatry clinic during study period. But this study focused indirect contact for psychiatry care, so which contact you assessed current or any contact for help sought? Please describe it clearly.

**Result section**

1. Other occupational status described as daily laborer, shoeshine, and prisoners. Is it being prisoner categorized as occupation? Modify this.

**Finally**; - thank you for your contribution to existing body of knowledge’s
