# Supplementary material for: Pathways to psychiatric care in Debre Berhan, Ethiopia: A cross-sectional study
Source: PLoS One. 2025 Jul 24;20(7):e0328724. doi: 10.1371/journal.pone.0328724 (PMC12289035; doi:10.1371/journal.pone.0328724)
Supplement: Attachment — Submitted filename: Response_to_Reviewers.docx [file pone.0328724.s006.docx]

# Cover Letter

Date: 20-06-25

**Prof. Alemayehu Molla Wollie**

**Editor-in-Chief**

**PLOS ONE**

**Re: Pathways to Psychiatric Care in Debre Berhan, Ethiopia: a cross-sectional study, PONE-D-24-27256R1**

**Dear Prof. Alemayehu,**

We are grateful for the thorough review of our manuscript and for the insightful comments for the second time. We have now made changes to the manuscript based on the comments. We also presented our point-by-point responses to the editor and reviewers' comments.

Thank you again for considering our manuscript for publication in your journal.

Kind regards,

Kaleab Berhanu, on behalf of the authors

**Response to the Editor’s Comment:**

I appreciate that you have revised your manuscript accordingly. Since the first reviewers were unavailable, I invited new reviewers to the revised version. They have also considered review reports of original reviewers, and they are satisfied with your revised version despite minor comments below. Please also clarify ethical issues while interviewing people taking care of their mental illness. I am not sure only oral consent is ethically enough to interview study participants. Once you address this issue and the minor comments of one reviewer, I hope your manuscript will be accepted for publication.

*Thank you for your comment. As stated in the methods section page 8 line 136-140, we assessed participants' capacity to give consent using a three-item tool to evaluate their ability to give consent before enrollment. Only those who were capable of providing consent were included in the study. Given the minimal risk involved and the nature of the study, we assumed that oral consent was sufficient. The other reason we went for oral consent was that we expected participants to be less educated and have limited ability to read and write, making it challenging to manage written consent procedures (one-third of the study participants are not able to read and write). To ensure consistency and inclusiveness, we chose to obtain oral informed consent after confirming the participant’s capacity to participate in the study. All participants were fully informed about the study’s purpose, their rights, and the voluntary nature of participation. This approach was approved by the Debre Berhan University College of Health Sciences ethical review committee.* *The meeting and protocol numbers referenced in the ethical committee letter were 1/2021 and 11/12/CHS/SPH.*

**Response to the Reviewer’s Comment:**

**Response for reviewer one (Tamene Berhanu Alaho (Mental Health Specialist)) comments**

**Overall:** I would like to thank for giving chance to review this interesting work. This study will have great value in low and middle income countries to enhance awareness and prevention strategies for mental health wellbeing. This manuscript reviewed strongly by previous reviewers and authors revised manuscript based on comments. I hope this is good job.

I have few minor comments, find it below

**Comments**

**Abstract section**

1. Describe the meaning of pathways to psychiatric care and its impact on mental health

*Thank you for the comment. We have added the meaning of pathways to psychiatric care and its impact on mental health. Please refer to page 2, lines 17–21 of the revised manuscript (also reported below).*

“Pathways to care are the steps individuals went through before finally consulting formal psychiatric services. In developing countries, people with mental disorders (PWMDs) often first consult traditional or religious healers, which may delay treatment. Although studies from different part of Ethiopia confirm this trend, factors influencing indirect pathways remain insufficiently explored.”

**Introduction/Background**

1. It is better to explain in detail about indirect Pathways to psychiatric care challenges and its contribution to mental disorders.

*Thank you for the comment. We have now included an explanation of indirect pathways to psychiatric care, the challenges associated with them, and their contribution to mental health outcomes. Please refer to page 4, lines 53–60 and page 5, lines 65-71 of the revised manuscript (also reported below).*

“People with different kinds of mental disorders follow different roots in receiving mental health care. Those events, processes, and intervals before medical treatment are called pathways to care (3). Pathways usually begin at some identifiable point in the social structure, prompted by the culturally mediated help-seeking interaction between the distressed person and their significant others (4). Pathways to psychiatric care could be direct or indirect. Direct care is a care provided by a psychiatrist or mental health professionals with no alternative sources. On the contrary, indirect care is a care provided by psychiatrists or mental health professionals after utilizing alternative sources.”

“One of the challenges of utilizing indirect pathways to care is delaying care. In low-income countries, most PWMDs had a longer duration of untreated illness (12, 13). Some of the reasons for this are prioritizing traditional and faith healers as primary sources of help (14), poor mental health infrastructure (15), lacking knowledge about mental health service availability (16), and considering mental illness as trivial (17). Delay in receiving psychiatric care can lead to an increased level of distress or disability and an increased burden in early detection, identification, and intervention of mental disorders (18, 19).”

2. This study mentioned that ‘‘Debre Berhan) is known for the availability of different holy water places, which are considered an alternative source for mental health care. We expect most PWMDs from various parts of the country to visit those places, hoping for a cure’’. What is relevancy for this statement? Holy water place is easily available in most part of Ethiopia. Why various parts of Ethiopian people going to Debre Berhan for hoping cure? Since holy water is similar in any place. How holy water in Debre Berhan is superior than others place to think most part of country’s people to hope cure. Please remove this statement since holy water is common in all regions in Ethiopia, there is no clear evidence for superiority of holy water in different region.

*Thank you for bringing this to our attention. We have now removed those descriptions since they might mislead readers.*

3. Introductions section is bulky. It is better to make short and precise this section.

*Thank you for this specific comment. The previous reviewers asked to provide detailed descriptions of the concept of the study and made the introduction longer. Now, we have made some changes to shrink it to three pages. Please refer to the introduction section for changes we made.*

**Method**

1. Participants for this study from patients receiving care in psychiatry clinic during study period. But this study focused indirect contact for psychiatry care, so which contact you assessed current or any contact for help sought? Please describe it clearly.

*We apologize for not making what we considered an indirect path clearer. We considered indirect pathways as any contact for the current problem before visiting Debre Berhan Comprehensive Specialized Hospital (DBCSH). Please see page 9, lines 155-161 of the revised manuscript (also reported below).*

“Considering the recommendation of the WHO encounter form for developing countries, we have classified those participants who received psychiatric care directly from modern health care service providers (such as mental health professionals, general practitioners, primary health care providers, and others) for their current mental health disorder as those considered direct path. On the contrary, we classified those participants who received psychiatric care at the DBCSH psychiatric department after visiting alternative sources such as religious healers, traditional healers, herbalists, or other informal sources as those who utilized indirect paths.”

**Result section**

1. Other occupational status described as daily laborer, shoeshine, and prisoners. Is it being prisoner categorized as occupation? Modify this.

*Thank you for the comment. DBCSH provides mental health services to individuals in prison, and in our study, we categorized them under occupation as "prisoners" since they were incarcerated at the time of data collection. Participants respond, “I am a prisoner” when we ask them about their occupation; perhaps it would be better to consider their previous occupation before imprisonment.*

**Finally**; - thank you for your contribution to existing body of knowledge’s

*We also would like to thank you for your comments, which provided us with insightful points we overlooked.*
