# Supplementary material for: Pathways to psychiatric care in Debre Berhan, Ethiopia: A cross-sectional study
Source: PLoS One. 2025 Jul 24;20(7):e0328724. doi: 10.1371/journal.pone.0328724 (PMC12289035; doi:10.1371/journal.pone.0328724)
Supplement: Attachment — Submitted filename: Reviewer comment on pathway to psychiatric care.docx [file pone.0328724.s002.docx]

**Dear Authors,**

The study you undertake provides valuable insights into the factors influencing pathways to psychiatric care in Debre Berhan, Ethiopia. The authors' comprehensive analysis of sociodemographic characteristics, perceived stigma, diagnosis, and help-seeking behaviors contributes to a better understanding of the challenges faced by individuals with mental health disorders in accessing appropriate care. Below are some concerns you need to go through and react

**Introduction part**

**Concerns**

- **Line 102-103:** You mention that little is known about pathways to psychiatric care in Ethiopia, especially indirect pathways. Could you elaborate on the research gap and why this is a significant area to investigate?
- **Line 111-112:** How might the availability of holy water places influence the decision-making process of PWMDs in Ethiopia? Are there any specific beliefs or practices associated with these places that might impact their choice of care?
- **Line 116-118:** How did the WHO pathways to psychiatric care encounter form contribute to the data collection and analysis process? What specific insights did it provide that might not have been possible with other methods?

**Method part**

**Concerns**

- **Line 134:** Could you elaborate on the reason for using a 3% margin of error and a 95% confidence interval? Were these values chosen based on specific guidelines or conventions in your field?
- **Line 138:** How was the expected number of PWMDs receiving psychiatric care at DBCSH during the study period obtained? Was this based on previous data, projections, or other sources?
- **Line 140:** Please provide more details about the non-response rate used for the final sample size calculation. How was this rate determined?
- **Line 142:** Can you explain why consecutive sampling was the choice for your research, considering its non-probability characteristics?
- **Line 145:** Could you elaborate on the three-item questionnaire used to assess participant capacity to consent? What were the specific questions asked?
- **Line 147:** Given the potential for bias in verbal consent, consider discussing any measures taken to ensure the validity and reliability of the consent process.
- **Line 159:** Given that the WHO encounter form has been used in previous studies in Ethiopia, what are the specific challenges or limitations that you anticipate encountering in using this tool in your study?
- **Line 174:** Could you provide more details about the specific previous studies that you adapted the questionnaires from for assessing social-related factors? How did you ensure that the adapted questionnaires were appropriate and valid for your study population?
- **Line 187:** Why did you choose to develop your questionnaire to assess clinical-related characteristics, rather than using existing validated instruments? What were the specific factors that you wanted to measure that were not covered by the WHO encounter form?
- **Line 193:** Could you provide more information about the specific items from the SRQ-20 that you included in your study? How did you ensure that these items were relevant and appropriate for assessing comorbid somatic symptoms in your population?
- **Line 200:** What specific mental health conditions were included in the assessment of mental health literacy?
- **Line 202:** Can you provide more details about the WHO pathways to psychiatric care encounter form and how it differs from the ten-item questionnaire?
- **Line 203:** Please elaborate on the specific studies from Ethiopia (references 13 and 29) that were used to adapt the ten-item questionnaire. What were the key similarities and differences between those studies and your research?
- **Line 204:** How did you ensure the ten-item questionnaire adequately assessed participants' perceptions of the cause, treatment, and curability of mental illness? Were there any pilot testing or validation procedures conducted?

**Result part**

- In **lines 222-225**, you report a high response rate of 98.2% among PWMDs. Could you please provide more details about the characteristics of the non-respondents (e.g., age, gender, disability type) to assess potential biases in your sample? Additionally, could you elaborate on the reasons for the interruptions and unwillingness to participate, as this may shed light on factors influencing participation rates?
- **Line 285**: Could you elaborate on why the adjusted odds ratios for significantly associated variables are substantially higher than their crude counterparts?

**Discussion part:**

- **Lines 423-424**: Could the authors elaborate on the potential mechanisms through which perceived stigma, family influence, and lack of access contribute to the decision to utilize indirect pathways?
- **Line 445**: Given the prominent role of religious healers in this study, how do the authors envision integrating these practices with modern psychiatric services to improve access to care?
- **Lines 443-447**: Beyond the recommendations for collaboration between mental health professionals and religious organizations, what other policy interventions could be implemented to address the challenges identified in this study?

**Better add the followings**

1. Study strengths and limitations

2. Recommendation
